# Sleep Efficiency May Predict Depression in a Large Population-Based Study

**DOI:** 10.3389/fpsyt.2022.838907

**Published:** 2022-04-13

**Authors:** Bin Yan, Binbin Zhao, Xiaoying Jin, Wenyu Xi, Jian Yang, Lihong Yang, Xiancang Ma

**Affiliations:** ^1^Department of Clinical Research Center, The First Affiliated Hospital of Xi'an Jiaotong University, Xi'an, China; ^2^Department of Psychiatry, The First Affiliated Hospital of Xi'an Jiaotong University, Xi'an, China

**Keywords:** depression, polysomnography, SHHS, men, sleep efficiency

## Abstract

**Objectives:**

The purpose of our study was to investigate the effect of objective sleep characteristics on the incidence of depression.

**Methods:**

The participants of our study (1,595 men and 1,780 women with 63.1 ± 10.7 years) were selected from the Sleep Heart Health Study (SHHS) datasets. Depression was defined as the first occurrence between SHHS visit 1 and visit 2. Objective sleep characteristics, including sleep efficiency (SE), wake after sleep onset (WASO), sleep fragmentation index (SFI) and arousal index (ArI), were monitored by polysomnography. Multivariable logistic regression was used to explore the relationship between sleep characteristics and depression.

**Results:**

A total of 248 patients with depression (7.3%) were observed between SHHS visits 1 and 2. After adjusting for covariates, SE (odds ratio [OR], 0.891; 95% confidence interval [CI] 0.811–0.978; *P* = 0.016) and WASO (OR, 1.021; 95% CI 1.002–1.039; *P* = 0.026) were associated with the incidence of depression. Moreover, the relationship between SE and depression was more pronounced in men (OR, 0.820; 95% CI 0.711–0.946; *P* = 0.007) than in women (OR, 0.950; 95% CI 0.838–1.078; *P* = 0.429) in subgroup analysis (*P*_interaction_ < 0.05).

**Conclusions:**

SE and WASO may be markers for the incidence of depression. The association between SE and depression was intensified in men.

## Introduction

Depression, characterized by feelings of sadness, slowed thinking, and loss of interest, is one of the main causes of disability and the overall disease burden around the world (1). The number of global depression cases increased from 172 million in 1990 to 258 million in 2017 (2). The common risk factors for depression include serious or chronic diseases, personal or family histories of depression, and traumatic or stressful events (3). Sleep is also a useful marker for the occurrence of depression (4).

Previous observational studies have demonstrated a bidirectional relationship between sleep disorders and depression (5, 6). Insomnia is a common syndrome, and predictor of depression (7, 8). Sleep architecture such as REM sleep was closely related to depression (9, 10). An increased percentage of fragmented REM sleep was also correlated with depressive symptoms in adolescents (11). Moreover, objective sleep characteristics such as sleep efficiency (SE) and wake after sleep onset (WASO) were associated with human health, but little evidence was found about the association between objective sleep characteristics and depression (12, 13).

Polysomnography (PSG) is usually employed to monitor sleep characteristics such as SE, WASO, sleep fragmentation index (SFI), and arousal index (ArI) in total sleep, REM sleep and non-REM (NREM) sleep. The aim of our study was to investigate the effect of objective sleep characteristics on the incident of depression based on PSG records.

## Materials and Methods

### Study Population

Data on the participants in the present study was obtained from the Sleep Heart Health Study (SHHS). The SHHS (ClinicalTrials.gov Identifier: NCT00005275) was a dataset consisting of nine existing “parent” cohort studies including the Framingham Offspring Cohort, the Hagerstown and Minneapolis/St. Paul sites of the Atherosclerosis Risk in Communities study, the Hagerstown, Sacramento, and Pittsburgh sites of the Cardiovascular Health Study, the Strong Heart Study sites in South Dakota, Oklahoma, and Arizona; and studies of respiratory disease in Tucson and of hypertension in New York. There was a clear data quality assurance and control system for the SHHS. All participants provided written informed consent, and the study was approved by the ethical review board at each site (https://doi.org/10.25822/ghy8-ks59). Between 1995 and 1998, SHHS visit 1 collected information about in-home PSG records, demographics, sleep habit questionnaires, and medical histories. SHHS visit 2 was performed between 2001 and 2003. In this study, individuals were excluded if they had (1) missing data at follow-up (*n* = 777), (2) previous depression (*n* = 420), (3) missing depression status (*n* = 433), and (4) not followed at SHHS visit 2 (*n* = 799). Finally, 3,375 participants were included in the study.

### Objective Sleep Characteristics

Objective sleep characteristics including SE, WASO, SFI, ArI-Total, ArI-REM, and ArI-NREM were monitored by overnight in-home PSG (P-Series; Compumedics, Abbotsville, Australia). The PSG was set up by a team that included a sleep technician and an assistant. PSG records were obtained in an unattended environment. The next morning, a technician returned to the participant's home at a Pre-arranged time to collect the sleep monitors. If the record is insufficient, a repeat PSG monitor request is initiated. The entire monitoring process tried to keep the participants consistent with their usual sleep. SFI refers to the number of awakenings plus sleep-stage shifts divided by total sleep time. SE is calculated as the total sleep time divided by the time in bed. WASO is defined as the period of wakefulness that start from first falling asleep to being fully awake and no attempt to go back to sleep. The ArI is calculated by dividing the number of arousals by the total sleep time (14). Apnea-hypopnea index (AHI) was defined as the total number of apnea and hypopnea occurrences divided by the total sleep time (accompanied by at least a 4% drop in oxygen saturation). Sleep duration using in this study was defined as the total sleep time during the PSG records. Total time in bed was referred to the time from lights off to lights on, rounded to nearest minute.

### Outcome

Depression status was based on the diagnosis and medication history of the “parent” cohort study. All depression medications were recorded during the interview. Medication information was later categorized by a physician review. Depression was defined as the first occurrence between SHHS visit 1 and 2 in this study.

### Covariates

Information about age, sex, race, education, marital status, smoking status, alcohol use, benzodiazepine use, sleep duration, history of common disease (including obesity, hypertension, and diabetes mellitus), and history of major cardiovascular disease (including myocardial infarction, heart failure, and stroke) were obtained at baseline.

### Statistical Analysis

Baseline characteristics are presented as mean (SD) for continuous variables and number (percentage) for categorical variables. Chi-square tests and independent sample *t*-tests were employed to detect the difference between the depression and control groups. Multivariable logistic regression was used to examine the effect of each individual sleep parameter (SFI, SE, WASO, ArI-Total, ArI-REM, and ArI-NREM) on the incident of depression. The results were reported with odds ratios (ORs) and 95% confidence intervals (CIs). Each individual sleep parameter was adjusted for age, sex, race, education, marital status, smoking status, alcohol use, benzodiazepine use, sleep duration, history of common disease, and history of major cardiovascular disease in multivariable logistic regression analysis. Based on multivariable logistic regression analysis, subgroup analysis was performed to further explore the association between objective sleep characteristics and depression stratified by sex (men vs. women). All statistical analyses were conducted using SPSS (version 24.0; SPSS Inc., Chicago, IL). A two-sided *P* value of < 0.05 was considered significant.

## Results

### Baseline Characteristics

A total of 248 patients with depression were observed between SHHS visit 1 and 2. Depressive patients had more women and benzodiazepine use than the controls. Individuals with depression also had relatively low levels of education. In addition, patients with depression were prone to have decreased SE and increased WASO. The baseline characteristics of the patients with and without depression are shown in Table 1.

**Table 1:** Subject characteristics in participants with or without depression.

**Variables**	**All (*n* = 3,375)**	**Depression (*n* = 248)**	**Non-depression (*n* = 3,127)**	***P* value**
Age, years	63.1 ± 10.7	63.1 ± 11.4	63.1 ± 10.6	0.967
Sex, *n* (%)				0.011
Men	1,595 (47.3)	98 (39.5)	1,497 (47.9)	—
Women	1,780 (52.7)	150 (60.5)	1,630 (52.1)	—
Race, *n* (%)				0.709
White	2,941 (87.1)	218 (87.9)	2,723 (87.1)	—
Other	434 (12.9)	30 (12.1)	404 (12.9)	—
Education, *n* (%)				0.003
≤ 15 years	1,936 (63.2)	162 (72.3)	1,774 (62.4)	—
>15 years	1,129 (36.8)	62 (27.7)	1,067 (37.6)	—
Marital status, *n* (%)				0.083
Married	2,682 (80.7)	185 (76.4)	2,497 (81.0)	—
Other	642 (19.3)	57 (23.6)	585 (19.0)	—
Smoking status, *n* (%)				0.845
Current smoker	319 (9.5)	26 (10.5)	293 (9.4)	—
Former smoker	1,469 (43.6)	106 (42.7)	1,363 (43.7)	—
Never smoker	1,579 (46.9)	116 (46.8)	1,463 (46.9)	—
Alcohol use, *n* (%)				0.240
At least 1 drink per day	1,423 (45.1)	94 (41.4)	1,329 (45.4)	—
None	1,729 (54.9)	133 (58.6)	1,596 (54.6)	—
Benzodiazepine use, *n* (%)	128 (3.8)	23 (9.3)	105 (3.4)	<0.001
History of common disease, *n* (%)				
Obesity	1,047 (31.0)	86 (34.7)	961 (30.7)	0.196
Hypertension	1,538 (45.6)	128 (51.6)	1,410 (45.1)	0.047
Diabetes mellitus	215 (6.4)	18 (7.3)	197 (6.3)	0.552
History of major CVD, *n* (%)				
Myocardial infarction	196 (5.8)	20 (8.1)	176 (5.6)	0.071
Heart failure	51 (1.5)	6 (2.4)	45 (1.4)	0.343
Stroke	82 (2.4)	7 (2.8)	75 (2.4)	0.676
Objective sleep characteristics				
SE, %	83.4 ± 9.9	81.9 ± 10.8	83.5 ± 9.9	0.018
WASO, min	60.1 ± 42.1	64.2 ± 42.9	59.7 ± 42.1	0.107
SFI, events/h	8.8 ± 3.2	9.0 ± 3.3	8.8 ± 3.2	0.326
ArI-Total, events/h	19.0 ± 10.3	18.3 ± 9.2	19.1 ± 10.4	0.260
ArI-REM, events/h	15.1 ± 10.6	14.3 ± 9.8	15.2 ± 10.7	0.183
ArI-NREM, events/h	19.9 ± 11.2	19.2 ± 10.0	19.9 ± 11.3	0.285
AHI, events/h	10.0 ± 13.1	9.3 ± 10.5	10.0 ± 13.2	0.269
Sleep duration, min	365.9 ± 61.2	363.2 ± 68.8	366.1 ± 60.6	0.527
Time in bed, min	439.3 ± 56.3	442.0 ± 56.9	439.1 ± 56.3	0.423

*AHI, apnea hypopnea index; ArI, arousal index; 95% CI, 95% confidence interval; CVD, cardiovascular disease; NREM, Non-rapid eye movement; OR, odds ratio; REM, rapid eye movement; SE, sleep efficiency; SFI, sleep fragmentation index; WASO, wake after sleep onset*.

*Results are presented as mean ± standard deviation or n (%). The P values represent the difference between two groups*.

### Sleep Characteristics and Depression

The multivariable logistic regression analysis showed that SE (OR, 0.891; 95% CI 0.811–0.978; *P* = 0.016) and WASO (OR, 1.021; 95% CI 1.002–1.039; *P* = 0.026) were significantly associated with the incidence of depression (Table 2). No significant associations of SFI, ArI-Total, ArI-REM, and ArI-NREM with incident of depression were found.

**Table 2:** ORs and 95% CIs for sleep characteristics associated with depression.

	**Univariate models**		**Multivariable adjusted^a^**		**Multivariable adjusted^b^**	
Sleep characteristics ^*^	**OR (95% CI)**	***P***	**OR (95% CI)**	***P***	**HR (95% CI)**	***P***
SE	0.930 (0.876 to 0.988)	0.019	0.887 (0.808 to 0.974)	0.012	0.891 (0.811 to 0.978)	0.016
WASO	1.012 (0.977 to 1.027)	0.108	1.021 (1.003 to 1.040)	0.022	1.021 (1.002 to 1.039)	0.026
SFI	1.103 (0.907 to 1.341)	0.326	1.156 (0.929 to 1.439)	0.195	1.156 (0.929 to 1.439)	0.194
ArI-Total	0.962 (0.899 to 1.029)	0.260	0.977 (0.907 to 1.053)	0.547	0.979 (0.909 to 1.055)	0.577
ArI-REM	0.956 (0.895 to 1.022)	0.183	0.972 (0.905 to 1.043)	0.429	0.975 (0.908 to 1.046)	0.480
ArI-NREM	0.967 (0.909 to 1.028)	0.285	0.980 (0.915 to 1.049)	0.559	0.981 (0.916 to 1.050)	0.579
AHI	0.995 (0.985 to 1.006)	0.365	0.980 (0.922 to 1.041)	0.512	0.979 (0.921 to 1.041)	0.499

*AHI, apnea hypopnea index; ArI, arousal index; 95% CI, 95% confidence interval; NREM, Non-rapid eye movement; OR, odds ratio; REM, rapid eye movement; SE, sleep efficiency; SFI, sleep fragmentation index; WASO, wake after sleep onset*.

* *Per 5-unit increased*.

a *Each individual sleep parameters was adjusted for age, sex, race, education, marry status, smoking status, alcohol use, benzodiazepine use and sleep duration*.

b *Adjusted for a+ history of common disease and history of major cardiovascular disease*.

### Subgroup Analysis

**Figure 1:**
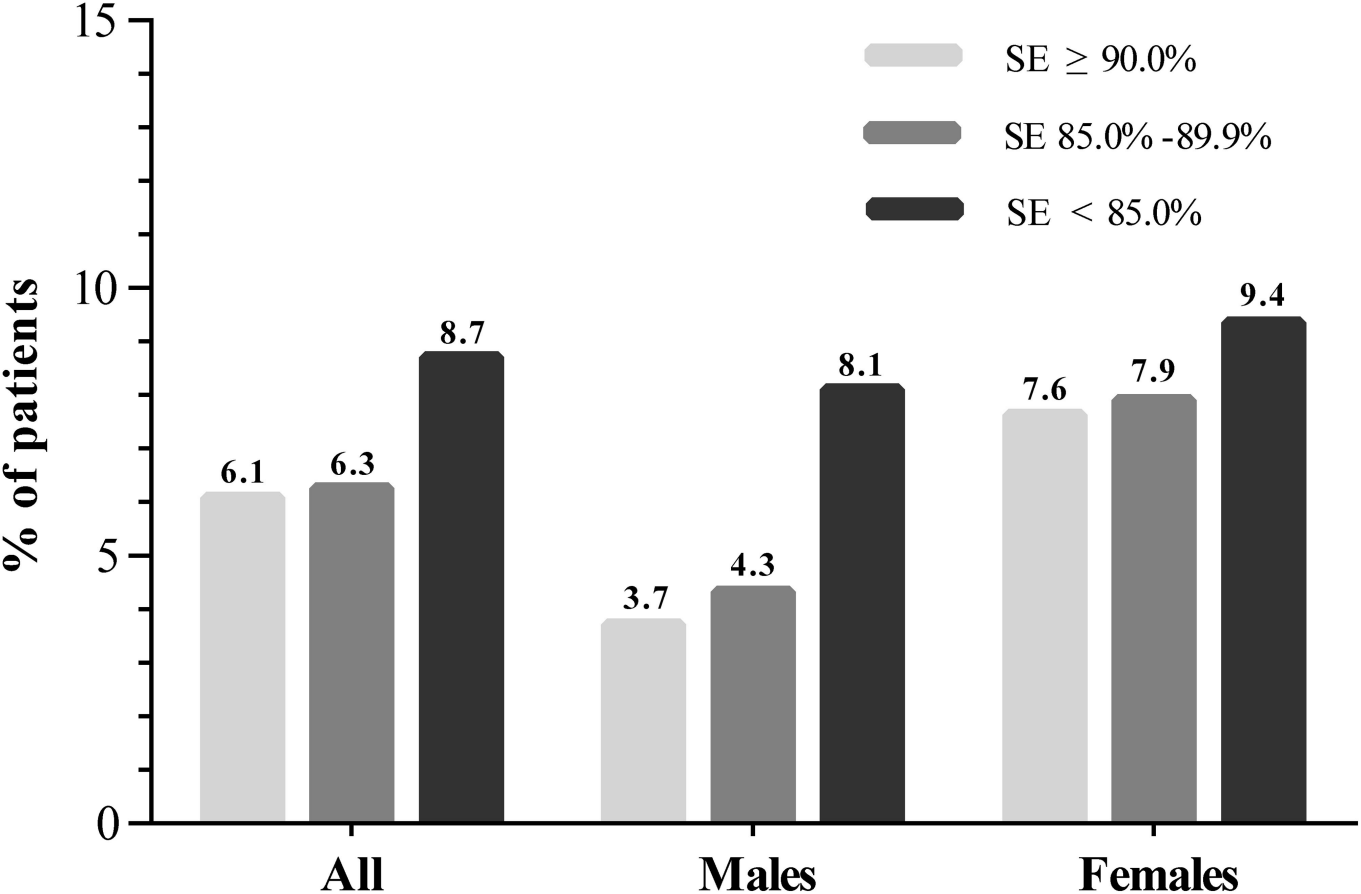
The distribution of incident depression in different SE categories.

We further conducted a subgroup analysis stratified by sex to investigate the effect of sleep characteristics on depression risk (Table 3). A significant interaction stratified by sex was found when exploring the association between SE and depression (*P*_interaction_ = 0.048). The incidence of depression among the participants with SE ≥ 90%, SE 85.0–89.9% and SE < 85.0% were 3.7, 4.3, and 8.1% (*P* = 0.003) in men and 7.6, 7.9, and 9.4% (*P* = 0.478) in women, respectively (Figure 1). After multivariable logistic regression analysis, increased SE was associated with a reduced risk of incident of depression in men (OR, 0.820; 95% CI 0.711–0.946; *P* = 0.007), but the association was not found in women (OR, 0.950; 95% CI 0.838–1.078; *P* = 0.429). Furthermore, no significant interaction was found when subgroup analysis was stratified by sex in the relationship between other sleep parameters and depression.

**Table 3:** Multivariate logistic regression analysis for sleep characteristics associated with depression stratified by sex.

	**Men**		**Women**		
Sleep characteristics ^*^	**OR (95% CI)**	***P***	**OR (95% CI)**	***P***	**P_**interaction**_**
SE	0.820 (0.711 to 0.946)	0.007	0.950 (0.838 to 1.078)	0.429	0.048
WASO	1.033 (1.007 to 1.060)	0.013	1.009 (0.983 to 1.035)	0.505	0.084
SFI	1.114 (0.821 to 1.511)	0.490	1.171 (0.852 to 1.609)	0.331	0.887
ArI-Total	0.975 (0.883 to 1.077)	0.622	0.978 (0.872 to 1.096)	0.699	0.803
ArI-REM	0.908 (0.812 to 1.016)	0.093	1.027 (0.934 to 1.130)	0.577	0.115
ArI-NREM	0.987 (0.903 to 1.079)	0.778	0.967 (0.869 to 1.075)	0.532	0.555
AHI	0.956 (0.880 to 1.039)	0.292	1.011 (0.922 to 1.108)	0.820	0.521

*AHI, apnea hypopnea index; ArI, arousal index; 95% CI, 95% confidence interval; NREM, Non-rapid eye movement; OR, odds ratio; REM, rapid eye movement; SE, sleep efficiency; SFI, sleep fragmentation index; WASO, wake after sleep onset*.

* *Per 5-unit increased*.

*Each individual sleep parameters was adjusted for age, sex, race, education, marry status, smoking status, alcohol use, benzodiazepine use and sleep duration, history of common disease and history of major cardiovascular disease*.

## Discussion

In the present study, we observed an association between sleep characteristics and the incident of depression. Our findings were based on SHHS, which is a large community-based population of middle-aged and elderly individuals. We provided evidence that decreased SE and long WASO were associated with an increased risk of depression. The association between SE and depression was more pronounced in men than in women in the subgroup analysis.

Several common factor, including restless leg syndrome, alcohol consumption, caffeine intake, poor sleep environment and chronic diseases such as bronchitis and angina pectoris, could cause sleep problem (15, 16). PSG and wrist actigraphy are typically used to evaluate objective sleep characteristics. In this study, the sleep characteristics including SFI, SE, WASO, ArI-Total, ArI-REM, and ArI-NREM were monitor by PSG records. Previous studies have found a close relationship between sleep fragmentation in REM and depressive syndrome in adolescents (11). However, we did not find a significant association of REM (ArI-REM) and NREM (ArI-NREM) with incident of depression in middle-aged and elderly individuals. The difference may be due to the different age groups used in the analysis. Circadian rhythms, sleep homeostasis and sleep-related hormone secretion are constantly changing during normal aging (17, 18). Previous study suggested that people with obstructive sleep apnea had higher prevalence of depression than general population (19). We also investigated the role of AHI in depression risk, but no significant association was found.

SE and WASO could reflect the general estimation of the overall quality of sleep (20). Our results show that for every 5% increase in SE, the incidence of depression will decrease by 12%. Moreover, every 5 min' increase in WASO will lead to a 2% increased risk of depression. Subgroup analysis was also performed to further explore the effect of sleep parameters on the incidence of depression. We found a significant difference between men and women in the association between SE and the incident of depression (*P*_interaction_ < 0.05). SE was found to be closely related to depression in men but not in women. Moreover, the relationship between WASO and depression was more pronounced in men than in women. Our findings indicate that more attention should be paid to individuals with poor SE in order to reduce the risk of depression, especially in men.

The mechanisms underlying the effects of SE and WASO on depression are not fully understood. SE and WASO were commonly used to measure objective sleep quality, while low SE is often accompanied by long WASO (21). Previous studies showed that low SE was associated with physical and psychological health in Chinese students (22). Additionally, individuals with low SE and long WASO were also found to have a high risk of major cardiovascular disease and chronic disease such as obesity, diabetes, hypertension, and coronary artery disease (13, 23, 24). Furthermore, poor SE may enhance microglial activation and aggravate neuro-inflammation, which may further increase the risk of depression (25–27).

This study had several strengths and limitations. We investigated the role of sleep characteristics in the incident of depression based on a relatively large community-based population. In addition, all sleep parameters were objectively monitored using in-home PSG. Depression was defined as the first occurrence between SHHS 1 and 2 visits. However, there was no information about the specific follow-up time of depression diagnosis; therefore, we used logistic regression analysis to explore the association between objective sleep characteristics and the incidence of depression. Moreover, the participants in the SHHS datasets were middle-aged and elder people; therefore, the findings may not be generalizable to younger individuals.

## Conclusion

We provided evidence that poor SE and long WASO may increase the risk of depression. The associations between poor SE and depression were more pronounced in men than in women. Improving sleep may help reduce the risk of depression.

## Data Availability Statement

The datasets presented in this study can be found in online repositories. The names of the repository/repositories and accession number(s) can be found below: https://doi.org/10.25822/ghy8-ks59.

## Ethics Statement

The studies involving human participants were reviewed and approved by Boston University Case Western Reserve University Johns Hopkins University Missouri Breaks Research, Inc. New York University Medical Center University of Arizona University of California at Davis University of Minnesota – Clinical and Translational Science Institute University of Washington. The patients/participants provided their written informed consent to participate in this study.

## Author Contributions

BY and XM raised the idea for the study. BY, BZ, XJ, WX, LY, and JY contributed to the study design, writing, and review of the report. BY and XM acquired the data in SHHS and BY participated in further data analysis. XM handled supervision in our study. All authors approved the final version of the report.

## Funding

This study was supported by the Natural Science Basic Research Program of Shaanxi (No. 2021JQ-395).

## Conflict of Interest

The authors declare that the research was conducted in the absence of any commercial or financial relationships that could be construed as a potential conflict of interest.

## Publisher's Note

All claims expressed in this article are solely those of the authors and do not necessarily represent those of their affiliated organizations, or those of the publisher, the editors and the reviewers. Any product that may be evaluated in this article, or claim that may be made by its manufacturer, is not guaranteed or endorsed by the publisher.
